# Molecular characterisation of 36 multilocus imprinting disturbance (MLID) patients: a comprehensive approach

**DOI:** 10.1186/s13148-023-01453-5

**Published:** 2023-03-01

**Authors:** Larissa Bilo, Eguzkine Ochoa, Sunwoo Lee, Daniela Dey, Ingo Kurth, Florian Kraft, Fay Rodger, France Docquier, Ana Toribio, Leonardo Bottolo, Gerhard Binder, György Fekete, Miriam Elbracht, Eamonn R. Maher, Matthias Begemann, Thomas Eggermann

**Affiliations:** 1 Medical Faculty, Institute for Human Genetics and Genome Medicine, RWTH Aachen University, Pauwelsstr. 30, 52074 Aachen, Germany; 2 Department of Medical Genetics, University of Cambridge, Cambridge, CB2 0QQ UK; 3 Stratified Medicine Core Laboratory NGS Hub, Department of Medical Genetics, University of Cambridge, Cambridge, UK; 4 MRC Biostatistics Unit, School of Clinical Medicine, University of Cambridge, Cambridge, UK; 5 The Alan Turing Institute, London, UK; 6 Pediatric Endocrinology, University Children’s Hospital, Universiy of Tuebingen, Tuebingen, Germany; 7 Department of Pediatrics, Semmelweis University, Budapest, Hungary

**Keywords:** Beckwith–Wiedemann syndrome, Silver–Russell syndrome, Multilocus imprinting disturbances, Maternal effect variants, ImprintSeq, MS-MLPA, Whole-exome sequencing

## Abstract

**Background:**

Imprinting disorders (ImpDis) comprise diseases which are caused by aberrant regulation of monoallelically and parent-of-origin-dependent expressed genes. A characteristic molecular change in ImpDis patients is aberrant methylation signatures at disease-specific loci, without an obvious DNA change at the specific differentially methylated region (DMR). However, there is a growing number of reports on multilocus imprinting disturbances (MLIDs), i.e. aberrant methylation at different DMRs in the same patient. These MLIDs account for a significant number of patients with specific ImpDis, and several reports indicate a central role of pathogenic maternal effect variants in their aetiology by affecting the maturation of the oocyte and the early embryo. Though several studies on the prevalence and the molecular causes of MLID have been conducted, homogeneous datasets comprising both genomic and methylation data are still lacking.

**Results:**

Based on a cohort of 36 MLID patients, we here present both methylation data obtained from next-generation sequencing (NGS, ImprintSeq) approaches and whole-exome sequencing (WES). The compilation of methylation data did not reveal a disease-specific MLID episignature, and a predisposition for the phenotypic modification was not obvious as well. In fact, this lack of epigenotype–phenotype correlation might be related to the mosaic distribution of imprinting defects and their functional relevance in specific tissues.

**Conclusions:**

Due to the higher sensitivity of NGS-based approaches, we suggest that ImprintSeq might be offered at reference centres in case of ImpDis patients with unusual phenotypes but MLID negative by conventional tests. By WES, additional MLID causes than the already known maternal effect variants could not be identified, neither in the patients nor in the maternal exomes. In cases with negative WES results, it is currently unclear to what extent either environmental factors or undetected genetic variants contribute to MLID.

**Supplementary Information:**

The online version contains supplementary material available at 10.1186/s13148-023-01453-5.

## Background

Imprinting disorders (ImpDis) consist of congenital diseases which are caused by dysregulation of genes underlying a monoallelic and parent-of-origin-specific expression. These so-called imprinted genes are organised in clusters, and their expression is regulated by imprinted differentially methylated regions (iDMRs)(for review: [1]). This methylation can be established during germline maturation (germline DMRs, gDMRs/primary DMRs), whereas in secondary DMRs it is often somatically acquired and dependent on hierarchical interactions with a neighbouring germline DMR.

ImpDis are caused by genomic alterations (copy number variations (CNVs), uniparental disomy (UPD), single nucleotide variants (SNVs)), as well as by aberrant methylation of ImpDis-specific iDMRs (imprinting defects, epimutations). More than ten ImpDis have been identified, and each of them is associated with specific iDMRs (for review: [2]). However, there is a growing number of patients with aberrant methylation of further imprinted loci in addition to the disease-specific ones. The highest frequencies of these multilocus imprinting disturbances (MLIDs) are observed in transient neonatal diabetes mellitus (TNDM1), Beckwith–Wiedemann syndrome (BWS), pseudohypoparathyroidism type 1b (PHP1b), and Silver–Russell syndrome (SRS) where they account for up to 50% of patients with specific imprinting defects [3]. The general prevalence of MLID is currently unknown as neither a common definition of MLID nor a standardised molecular assay exist. Furthermore, MLID testing is not routinely conducted in individuals with imprinting defects. The detection of MLID is additionally hindered by the mosaic distribution of imprinting defects [4], and by the clinical heterogeneity of MLID patients (e.g. [5, 6]).

The identification of MLID is currently mainly based on Methylation-specific Multiplex Ligation-dependent Probe Amplification (MS-MLPA), as this is the most frequently applied methodology in molecular diagnostics of ImpDis [7, 8]. Nevertheless, alternative techniques are available [8], and with ImprintSeq the first next-generation sequencing approach addressing specific imprinted loci has recently been developed [9].

The molecular mechanisms causing imprinting defects are widely unknown, but several genomic variants have already been identified to cause aberrant imprinting. They either comprise alterations within the iDMRs itself (*cis* acting factors, e.g. [10]), or variants in genes that are involved in the establishment and/or maintenance of genomic imprints in the oocyte and the (early) embryo. These *trans* acting variants either affect the foetal genome (e.g. *ZFP57, ZNF445*) or are so-called maternal effect genes which encode members of the subcortical maternal complex (SCMC). The SCMC is a large multiprotein complex consisting of NLRP2, NLRP5, NLRP7, PADI6, KHDC3L, TLE6, and OOEP and plays an important role in the proper oocyte maturation and early embryonic development (for review: [11, 12]). Consequently, genomic variants in these maternal effect genes are associated with female reproductive failure and pregnancy complications.

However, the large number of individuals with an ImpDis and MLID but without known genomic causes might indicate a role of environmental factors in the aetiology of MLIDs. In fact, several factors such as the parental metabolic and nutritional status, drug abuse, endocrine-disrupting substances, and in particular assisted reproductive technology (ART) have been suggested to affect the imprinting signatures (for review: [1]).

**Fig. 1:**
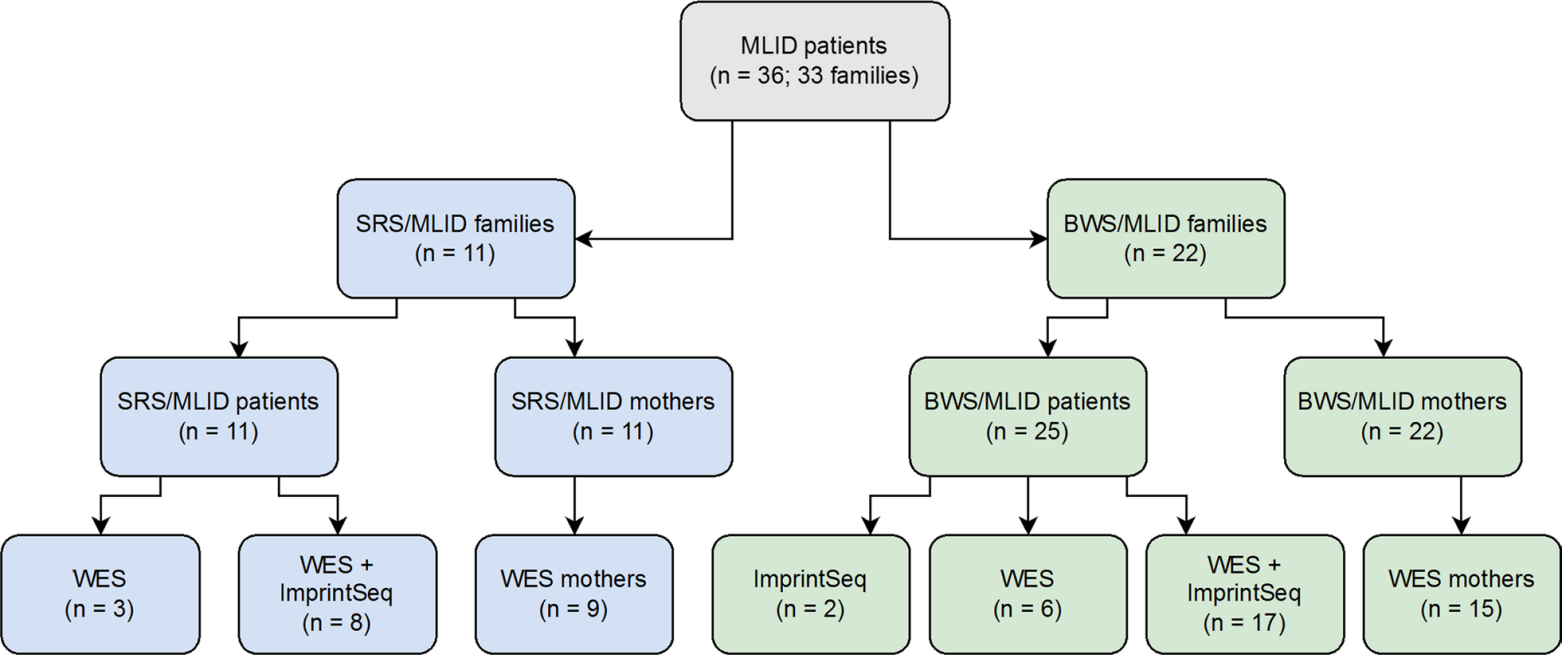
Study cohort. The study cohort consisted of eleven SRS/MLID patients and 25 BWS/MLID families. The latter included two sibpairs and a pair of concordant monozygotic twins. Due to the availability of DNA samples from the patients and their mothers, not all samples could be analysed by ImprintSeq and WES

Though several cases with MLID have been reported more than ten years after the publication of the first MLID family [13], homogeneous datasets comprising both genomic and methylation data are still lacking (Additional file 1: Table S1). Based on a cohort of 36 MLID patients (Fig. 1) from 33 families, we here present for the first time both methylation data obtained from next-generation sequencing (NGS) approaches and whole-exome sequencing (WES), thereby allowing a comprehensive molecular overview in this unique group of patients.

## Results

### MS-MLPA

**Fig. 2:**
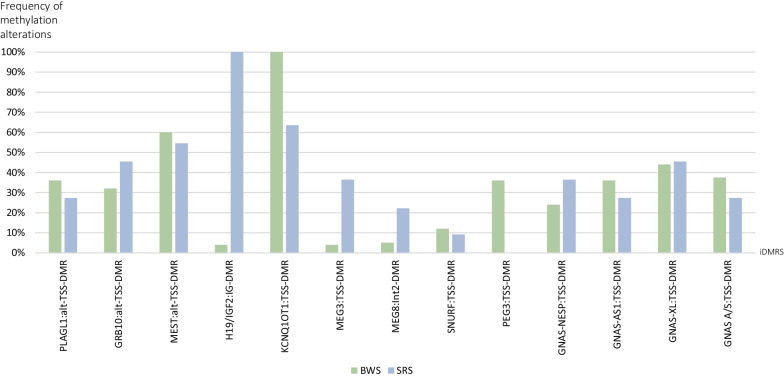
MS-MLPA results from the study cohort*.* By MS-MLPA, DNA methylation was determined at 13 iDMRs. With the exception of MEG8 and PEG3, all loci are associated with ImpDis. (green: BWS/MLID patients (*n* = 25), blue: SRS/MLID patients (*n* = 11))

MLID was diagnosed in 25 BWS patients with LOM at the IC2. Further methylation alterations were detected at up to eight iDMRs, with MEST and GNAS-XL being the most frequently affected loci (60.0% and 44.0%). PEG3 was the only iDMR affected by both LOM and GOM (Fig. 2; Additional file 1: Table S2).

In eleven SRS patients with LOM at the IC1, testing for further imprinted loci using the MS-MLPA assays ME032 and ME034 revealed a MLID. The MLID pattern comprised methylation disturbances at up to nine iDMRs per patient. The most frequently affected loci in addition to the IC1 were IC2 (63.6%) and MEST (54.5%). PEG3 was the only iDMR which was not affected in SRS/MLID (Fig. 2; Additional file 1: Table S2).

### ImprintSeq

LOM at the IC2 was confirmed in all 21 BWS/MLID patients tested by ImprintSeq. Their median methylation level (MML) at IC2 was 6.5, ranging from 0 to 26.0 (mean control: 45.7, SD: ± 4.5). MML values below 15 were observed in 20 cases with four patients showing complete LOM at IC2.

Additional methylation disturbances were observed at five to 30 further iDMRs per patient (median 18.2). In total, LOM was detected at 45 iDMRs, GOM at three iDMRs, and both GOM and LOM at two iDMRs.

**Fig. 3:**
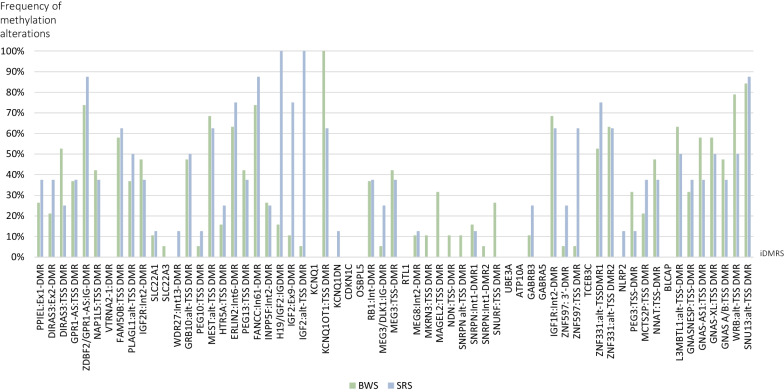
Frequency of significant methylation alterations per iDMR (3 SD confidence interval) obtained by ImprintSeq*.* (green: BWS/MLID patients (*n* = 19), blue: SRS/MLID patients (*n* = 8))

The non-ImpDis-associated iDMRs with the most frequent LOM were SNU13:alt-TSS-DMR (SNU13), and WRB:alt-TSS-DMR (WRB) (84.2% and 78.9% of BWS/MLID cases, respectively)(Fig. 3). The non-ImpDis-iDMR ZDBF2/GPR1 was most frequently affected by GOM with 73.7% of BWS/MLID cases.

In addition to the IC2, LOM was most frequently detected at MEST among the ImpDis-associated iDMRs (68.4%), followed by GRB10 and GNASA/B (47.4% each).

Comparing the mean MML of the controls of each iDMR with the mean MML of the BWS-MLID patients, a difference of 8.8 SD at IC2 was observed. Deviations of more than 4 SD were detected in ten further iDMRs with the GNAS-XL showing the most significant SD (6.67 SD) (Additional file 1: Table S3). In addition, a difference of 18.86 SD was shown for ZDBF2/GPR1.

In the eight SRS/MLID patients examined by ImprintSeq, LOM at IC1 was confirmed in all samples with a mean MML of 17.8, ranging from 0 to 39.8 (mean control: 56.1, SD ± 2.1).

Per patient, the number of iDMRs additionally affected by methylation disturbances ranged from six to 34. LOM was shown for 39 of the 63 examined iDMRs, GOM for six iDMRs.

In addition to LOM at the disease-associated locus IC1, the MEST and the IC2 iDMRs were the most frequently affected loci among ImpDis-associated iDMRs by LOM, (62.5% of cases each), followed by LOM at the GRB10 (50.0%).

Among the non-ImpDis-iDMRs, the most frequently hypomethylated DMRs were FANCC:In61-DMR (FANCC) and SNU13 (87.5% of SRS/MLID cases each). GOM mainly occurred at the ZDBF2/GPR1 and ZNF557:TSS-DMR (87.5% and 62.5% of SRS/MLID cases).

To assess the extent of LOM/GOM, the SRS/MLID patient’s mean MML of each iDMR was compared with the healthy control samples’ mean MML. As expected, the SRS/MLID patients’ mean MML at IC1deviated by 18.5 standard deviations (SD) from the healthy controls. Furthermore, a difference of 25.1 SD between the mean MMLs of controls and SRS/MLID patients at ZDBF2/GPR1 was observed. Discrepancies of more than 4 SDs were detected in 18 further iDMRs. The iDMR with the largest SD, apart from the IC1 and the ZDBF2/GPR1, was MEST (7.0 SD) (Additional file 1: Table S3).

**Fig. 4:**
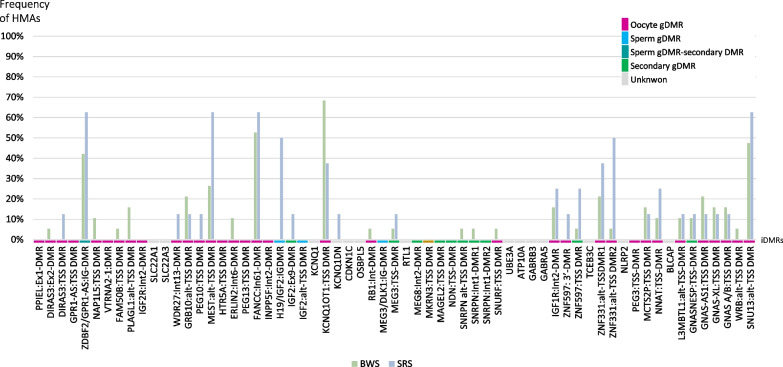
Frequency of high methylation alteration (HMAs) per iDMR (3 SD confidence interval) ascertained by ImprintSeq*.* Significant HMAs detected per iDMR with 3 SDs confidence interval and with a differential median methylation level above cut-off (0.185) in BWS/MLID (green, *n* = 19) and SRS/MLID patients (blue, *n* = 8)

The comparison of the frequencies of significant methylation alterations at individual loci between the BWS/MLID and SRS/MLID patients revealed that LOM at the BWS-associated IC2 was also frequently observed in SRS/MLID patients (62.5%), whereas LOM at the SRS-associated IC1 was only identified in 15.8% BWS/MLID patients. Discrepant frequencies among BWS/MLID and SRS/MLID could also be observed for further iDMRs (Fig. 4).

The ZNF597:TSS-DMR and ZNF331:alt-TSS-DMR (ZNF331) were more frequently affected in SRS (57.2% and 22.4%, respectively), whereas in BWS/MLD the MAGEL2:TSS-DMR (31.6%), WRB (28.9%), DIRAS3:Ex2-DMR (27.6%), SNURF (26.3%), and GNAS-AS (20.4%) were altered more often.

Comparing the extent of the alteration between the BWS/MLID and SRS/MLID patients, a difference of 7.0 SD and 4.9 SD was detected at the IC1 and ZDBF2/GPR1, respectively. For other loci, the mean MMLs of each iDMR were comparable between the two groups and derivate from each other by a maximum of 1.2 SD (Additional file 1: Table S3).

In both siblings with BWS/MLID of family 15, due to maternal biallelic variants in *PADI6* [16], ImprintSeq revealed LOM of IC2 as well as of PLAGL1, GRB10, MEST, MEG3, GNASA/B, and five non-ImpDis-iDMRs. However, the MMLs differ by up to 17.8 SD between the siblings.

In the monozygotic female twins of family 18 with clinically concordant BWS features (BWS-18a, BWS-18b), the methylation patterns were comparable. In both twins, the same 14 iDMRs showed aberrant imprinting. In addition, the MMLs of each iDMR differ by at most 2 SD between the twins, and 50 out of 63 iDMRs revealed a difference of ≤ 0.7 SD.

By using the cut-off of 0.185 [9], 359 of 509 (70.5%) detected significant methylation alterations with 3 SD were classified as high methylation aberrations (HMAs). The remaining significant LOMs/GOMs with a differential median methylation level (DMML) above the cut-off were classified as median methylation aberrations (MMAs). Comparing primary and secondary iDMRs, it was observed that 73.1% (310/424) of alterations in primary iDMRs were classified as HMA, whereas 58.7% (44/75) in secondary iDMR were HMAs.

To avoid overdiagnosis of MLID, Ochoa et al. [9] had proposed that the term MLID should be used in cases with a LOM/GOM HMA at an ImpDis-associated iDMR or with two LOM/GOM HMAs at non-ImpDis-iDMRs (in addition to the primary ImpDis-specific imprinting defect). The 27 patients examined by ImprintSeq in our study all met these criteria (Additional file 1: Table S4).

### Comparison between MS-MLPA and ImprintSeq

Since the nomenclature of iDMRs is inconsistent and the amount of interrogated CpG dinucleotides differs between several methods, it was ensured that the CpGs analysed by ImprintSeq were also targeted by the MS-MLPA probes (Additional file 1: Table S5). As a result, the data from ImprintSeq and MS-MLPA could be compared for 13 iDMRs (348 methylation sites). In general, the results from ImprintSeq were consistent with those of the MS-MLPA, but they differed in 22 methylation sites in ten patients (Additional file 1: Table S6). These discrepancies mainly comprised LOMs which were not detected by MS-MLPA but by ImprintSeq. Vice versa, GOM of PEG3 was identified by MS-MLPA but not by ImprintSeq. In the case of discrepant results, MS-MLPA was repeated, a different MS-MLPA kit or MS pyrosequencing was conducted. However, the discrepancies at these 22 sites were not resolved, but might be explained by the different assays.

### WES results

In the mothers of the MLID patients, the six previously published pathogenic variants in *NLRP2, NLRP5*, and *PADI6* were confirmed (Additional file 1: Table S7), but no further obviously pathogenic maternal effect variants could be detected.

Heterozygosity for the single nucleotide variant (SNV) rs200193926 in *NLRP7* was found in one patient (BWS-4; Additional file 1: Table S7). The variant chr19(GRCh38):g.54939205A > T, p.Phe538Leu) in exon 4 (NM_001127255.1: c.1614 T > A) was predicted to be deleterious by SIFT (https://sift.bii.a-star.edu.sg) and benign by MutationTaster (https://www.mutationtaster.org/), and the Phred score (https://cadd.gs.washington.edu) of this variant was 14.16. In ClinVar, it is listed as variant of uncertain significance (VUS) in a case with recurrent hydatidiform mole (VCV000893606.4), and its global minor allele frequency is 0.00040. Since DNA of the mother was not available, the alteration could not be classified as maternal effect variant.

WES analysis of the MLID patients was negative: neither in the MLID-causing genes *ZFP57* and *ZNF445* nor in another gene variants could be detected that might cause aberrant methylation at imprinted loci.

### Clinical findings

Clinical scoring was applied using the BWS spectrum (BWSp)-scoring system suggested by Brioude et al. [17] and the SRS Netchine-Harbison Clinical Scoring System (NH-CSS) [18], with a score of ≥ 4 criteria for BWS and at least 4/6 for SRS.

The BWS/MLID patients with clinical data displayed a BWSp-score between 3/20 and 11/20 (average 7.0), and the NH-CSS-score of the SRS/MLID patients for whom clinical data were available ranged from 2/6 to 5/6 (average 4.2) (Additional file 1: Table S8a).

Some atypical clinical features were documented in the BWS/MLID patients. Despite the clinical diagnosis of BWS, one patient was born small for gestational age (SGA; BWS-6) and in two patients growth retardation in childhood was reported (BWS-16 and BWS-17). Hyperphagia as a typical feature of PWS was reported once (BWS-17). In addition, feeding difficulties and protruding foreheads as characteristics of SRS were reported in five and two BWS/MLID patients, respectively (Additional file 1: Table S8b). Neurodevelopmental delay was recorded in 35.7% of BWS/MLID patients (*n* = 14) and 50.0% of SRS/MLID (*n* = 6), respectively.

The comparison of birth parameters revealed an average gestational age of 36.1 weeks for BWS/MLID and 35.5 weeks for SRS/MLID patients. In the case of BWS/MLID patients, birth height and weight were only slightly above the mean of the general population (weight: 0.51 SD score, and height: 0.50 SD score). In contrast, the average SD of the SRS/MLID patients’ birth weight was -2.60 and the birth length deviated by -2.83 SD (Additional file 1: Table S9).

### Maternal reproductive history

Data regarding the maternal reproductive history were available in 24 out of 33 families. In seven families (29.2%), either the mother herself and/or further close relatives had suffered from miscarriages. In three of these families, a pathogenic maternal effect variant had already been identified in the mother before [14–16]. Five patients were born after intracytoplasmic sperm injection (ICSI)/in vitro fertilisation (IVF) (19.2%), two patients after oocyte donation, and one patient after ovarian stimulation therapy (Additional file 1: Table S10).

## Discussion

An increasing number of reports indicate that MLID is a molecular overlapping finding in different ImpDis and can be accompanied by atypical clinical features in patients as well as by reproductive problems in the families (for review: [19]). However, a correlation between specific imprinting patterns and the clinical outcome is currently not obvious. Recent studies were hampered by small numbers of patients studied, by the lack of a consensus set of imprinted loci targeted by MLID studies and/or heterogeneous methodologies (Additional file 1: Table S1). With the recently developed NGS-based ImprintSeq assay [9], a robust and sensitive assay is now available for consistent methylation analysis. In contrast to available genome-wide methylation arrays, it enables the comparison with results from single-locus methylation-specific approaches as it specifically addresses iDMRs, including the currently known ImpDis-associated iDMRs.

Here, we report on the comprehensive molecular results obtained from a cohort of BWS/MLID and SRS/MLID patients by ImprintSeq [9] and by WES, in order to identify molecular differences which might explain the different clinical pictures of MLID patients.

The comparison of the aberrant methylation at single iDMRs between the BWS/MLID and SRS/MLID patients did not reveal obvious differences, with the exception of ZDBF2/GPR1. As expected, the SRS-specific IC1 LOM at the BWS-associated IC2 was also frequently observed in SRS/MLID patients, but the difference in the mean MML at this iDMR was marginal between the two groups.

By comparing the frequency of methylation alterations at individual loci, we observed that some iDMRs appeared to be more often affected in either the BWS/MLID or SRS/MLID patients, but the differences were not significant (Fig. 3; Additional file 1: Table S11).

The secondary ZDBF2/GPR1 was one of the iDMRs that was mostly affected by aberrant methylation in both syndromes. In nearly all cases, GOM was observed and demonstrated the largest deviation of the mean MML between MLID patients and healthy controls apart from IC1. This observation corresponds to that in BWS and SRS patients with isolated IC1 or IC2 LOM, respectively [9]. Notably, the GOMs in the single-locus cohort were mostly classified as MMAs [9], whereas the few alterations classified as HMAs were exclusively observed in MLID patients.

The contribution of aberrant methylation at the ZDBF2/GPR1 is interesting as mice with LOM of this secondary DMR (sDMR) show lack of ZDBF2 expression in the pituitary gland and the hypothalamus, resulting in reduced postnatal growth [20]. However, nearly all MLID patients exhibited GOM of this iDMR, a finding which is not consistent with the opposite growth phenotypes in SRS and BWS. Thus, a functional role of aberrant methylation at this locus is unlikely, but ZDBF2/GPR1 might be regarded as a region prone to aberrant imprinting. This observation is confirmed by reports from other ImpDis associated with MLID (SRS, BWS, PHP1b, Prader–Willi syndrome (PWS), Temple syndrome (TS14) [9, 21, 22]), whereas its imprint is normal in healthy controls [9]. However, since the current knowledge about ZDBF2 is based on mouse studies, it is unclear to what extent disturbed imprinting at the sDMR influences ZDBF2 levels and therewith growth in humans.

Further regions showing aberrant methylation in more than 60% of BWS/MLID and SRS/MLID were MEST, ERLIN2:Int6-DMR, FANCC, IGF1R:Int2-DMR (IGF1R), ZNF331, and SNU13 [23, 24]. These alterations have also been reported in MLID with other ImpDis [9, 23, 25–27]. As suggested for the ZDBF2/GPR1, this observation indicates that these iDMRs are particularly vulnerable and are therefore frequently affected in case of MLIDs. However, their contribution to the clinical features in MLID patients is rather uncertain, though for some loci an influence on growth has been postulated (MEST, IGF1R, ZNF331).

In 25% (6/24) of the tested MLID mothers putative pathogenic maternal effect variants were identified. The determination of the aberrant imprinting pattern of children in a family with *PADI6* mutations (BWS-15 [16]) confirmed the observation that *PADI6* associated MLIDs exhibit severe epigenotypes [9, 19, 28]. However, comparably severely aberrant MLID profiles were also detected in a patient from a *NLRP2* family (SRS-1), and in other patients without proven maternal effect gene variants (SRS-5, BWS-14). Nevertheless, the severity of the epigenotype might be influenced by the altered maternal effect gene and its role in embryonic development [10, 29]. Pathogenic variants in *PADI6* have been shown to arrest the development of the embryo at the four-cell stage [29]; therefore, these alterations might cause a more severe MLID episignature, whereas other SCMC factors function later in embryonic development. Interestingly, methylation analysis of the siblings of the *PADI6* family (BWS-15)[16] showed a heterogeneous MLID pattern. Both children showed methylation disturbances in six ImpDis-associated iDMRs and five non-ImpDis-iDMRs, but the extent of the deviations varied remarkably. This is accordance with the data from another family with maternal *PADI6* variants [24], indicating that the methylation profile of siblings does not appear to be consistent and results from a stochastic distribution of an error in imprint establishment and maintenance [30].

In contrast, an almost identical methylation pattern was identified in the monozygotic female twin pair with clinically concordant BWS features. Both the number of iDMRs with methylation disturbances and the extent were comparable between the twins, suggesting that the cause of MLID precedes the twinning process.

Various studies indicate an association between ART and an increased probability for children suffering from ImpDis (for review: [31–33]). Particularly for the aetiology of IC2 LOM in BWS, a causative role of ART has been suggested [33], presumably due to the vulnerability of this locus for environmental factors (e.g. [34]). The frequency of MLID patients born after ART in our cohort was higher than in the general population in developed countries (19.2% vs. 2%) [35]). Consistent with the observation of Lazaraviciute et al. [31], an association of ART with specific episignatures was not obvious in our cohort, indicating a stochastic occurrence of disturbances and iDMRs with the exception of the IC2.

Our data indicate that the “leading” phenotype in a MLID patient is determined by the most severely altered imprinted locus. The observation that the IC2 is the most vulnerable ImpDis-associated iDMR in case of MLID is in accordance with BWS as the leading phenotype in MLID. When both IC1 and IC2 iDMRs at 11p15.5 exhibit LOM, the phenotype appears to depend on the locus that is more severely affected, as suggested by the data from our five SRS/MLID patients with LOM at both iDMRs (Additional file 1: Table S3).

Several studies aimed to determine clinical differences between single-locus and MLID patients but have provided inconsistent results [30, 36, 37]. By comparing the birth parameters of the BWS/MLID and SRS/MLD patients with those of single-locus patients of previously published studies [36, 38], differences for both disorders became obvious. In patients ascertained as BWS, macrosomia at birth was less frequent in MLID patients in comparison with single-locus patients (MLID: weight: 0.51 SD, height: 0.50 SD vs. IC2 LOM: weight: 1.60 SD, height: 0.90 SD [38]). Notably, growth restriction at birth was less pronounced in SRS/MLID patients in our cohort than in single-locus cases (MLID: weight: − 2.60 SD, height: − 2.83 SD vs. single locus: weight: − 3.16 SD, height: − 4.51 SD [36]).

This is consistent with the hypothesis that effects of LOM at the primary iDMR epimutation at IC1 or IC2 are often accompanied by LOM at other loci with opposing effects on growth [37, 39].

Preterm delivery is a common feature at least for BWS [40]. In MLID, it occurs as well, and it is even more pronounced in this molecular subgroup than in BWS and SRS in general [36, 38] (BWS/MLID: average gestational age of 36.1; SRS/MLID: 35.5 vs. single-locus IC2 LOM: 36.4 [38]; IC1 LOM: 37.2 [36]).

Comparison of the clinical features between single-locus and MLID patients revealed some further differences. On average, the SRS/MLID patients met fewer NH-CSS criteria (4.17) than single-locus patients (5.86 [36]). In BWS/MLID, this comparison was hampered due to the lack of a common clinical scoring system in the past; thus, published clinical information is inconsistent. In addition, the frequencies of clinical features among single-locus patients vary remarkably in different studies [38, 41]. In accordance with Fontana et al. [42], the features nevus flammeus and polyhydramnios were reported more frequent in our BWS/MLID patients compared to single-locus patients (MLID: 83.3% and 40.0% vs single locus: 48.4% and 15.3%; Additional file 1: Table S8a). Notably, the frequency of nephromegaly/hepatomegaly (7.1%) was relatively low in our cohort, while lateralised overgrowth was detected with a higher frequency in MLID patients (61.5%).

In addition to the typical ImpDis features, some MLID patients showed further atypical clinical signs. In BWS/MLID, unusual clinical features ranged from feeding difficulties and growth retardation to hyperphagia, whereas we could not identify atypical clinical features in SRS/MLID patients. Furthermore, the association between MLID patients and an increased frequency of developmental delay (e.g. [4, 27]) was confirmed in our cohort (SRS/MLID: 50.0%; BWS/MLID: 35.7%). Finally, an excess of female patients was observed in our MLID cohort (66.7%). This is in agreement with the hypothesis that females are more prone to imprinting errors due to a reduced dosage of DNA methyltransferase 1 (DNMT1) which is responsible for the maintenance of X-chromosome inactivation, but also the maintenance of iDMRs methylation. The susceptibility of female embryos for MLID might thus be explained by the consumption of the DNTM1 during X-chromosome inactivation ([30]).

In order to identify genomic causes of MLIDs, WES was performed in MLID patients and their mothers. Among the 34 MLID patients analysed by WES, no pathogenic variant in a protein-coding gene could be identified. So far, only two genes have been reported in which biallelic pathogenic variants in the patients cause aberrant imprinting and ImpDis: Biallelic pathogenic variants in *ZFP57* can be detected in about half of TNDM1 patients, associated with hypomethylation at PLAGL1 and two other imprinted loci [43]. Recently, a homozygous *ZNF445* truncating variant has been suggested to cause aberrant methylation in TS14/MLID [44]. Our negative WES results in MLID patients confirm that pathogenic *ZFP57* variants are restricted to TNDM1/MLID patients [45] and that a common molecular cause in MLID patients does not exist, at least in protein-coding genomic regions. However, genomic variants predisposing for aberrant methylation might occur in noncoding regions and remain undetected by commonly used techniques such as WES.

In addition to the analysis of patients samples, we conducted WES in maternal DNA samples. In recent studies, homozygous and compound heterozygous pathogenic maternal effect variants in SCMC genes (*NLRP2, NLRP5, NLRP7, PADI6, KHDC3L, TLE6, OOEP, UHRF1*, and *ZAR1*) had been described (for review: [19]). In our cohort, we could confirm the already published variants in *PADI6, NLRP2*, and *NLRP5* in several families [14–16] but additional pathogenic variants were not detectable. WES analysis in the patient group revealed a VUS in *NLRP7* in one patient (BWS-4), but a maternal DNA sample was not available.

In contrast to pathogenic variants in *ZFP57* that are specifically associated with TNDM1 and a predictable imprinting signature, maternal effect variants appear to result in both different phenotypes and a considerable variability of affected imprinted loci [46]. Families with maternal effect variants are prone to children with MLID and reproductive problems such as recurrent miscarriages and hydatidiform moles [12]. Among the eight MLID families with miscarriages in our cohort, three mothers carry maternal effect variants (Additional file 1: Table S10). However, maternal effect variants which currently escape detection cannot be excluded. Future studies based on whole genome analysis will show whether additional disease-causing genes exist and whether the ratio of MLIDs resulting from pathogenic maternal effect variants is higher than the 25% determined in our cohort.

Several factors (e.g. parental metabolic and nutritional status, drug abuse, endocrine-disrupting substances, ART) have been discussed to have an impact on imprinting signatures (for review: [1]). Indeed, five patients in our cohort were born after ART (19.2%), supporting the assumption of an association between ART and BWS/MLID or SRS/MLID [33]. However, the precise role of environmental factors in the aetiology of MLID is difficult to assess. It therefore remains to be answered to what extent either further undetected genetic variants or environmental factors contribute to MLID.

## Conclusion

We here report for the first time a comprehensive set of molecular and clinical data in MLID patients with 11p15.5-associated imprinting disorders obtained by the same methodologies.

As the methylation data show, there is no evidence for a specific MLID episignature which might predispose to a specific modification of the BWS or SRS phenotype. This lack of epigenotype–phenotype correlation might be related to the mosaic distribution of imprinting defects and their functional relevance in specific tissues. As only 63 iDMRs were analysed, other commonly affected iDMRs might escape detection, but epigenome-wide array studies do not indicate additional iDMRs to be commonly affected as well (for studies see Additional file 1: Table S1).

Though the results from the NGS-based ImprintSeq assay were consistent with those of the MS-MLPA approach, NGS-based assays are generally more sensitive due to the larger number of targetable iDMRs as well as CpGs per iDMR. As MS-MLPA is an appropriate and broadly applied diagnostic tool, we suggest that ImprintSeq might be offered at reference centres in case of IC2-LOM-BWS or IC1-LOM-SRS patients with unusual phenotypes but MLID negative by MS-MLPA testing. Thus, patients currently tested negative for imprinting defects might be molecularly diagnosed by deep MS approaches like ImprintSeq in the future, with the additional chance to identify mosaic (epi)genetic constitutions.

An apparent cause for MLID was present in 25% of families with maternal effect variants. However, the pathogenicity prediction of these SCMC variants was not always unambiguous, and thus, the contribution of this type of molecular alterations to MLID requires further studies. Variants predisposing for MLID in the patients’ genome were not detected by WES in our cohort and are presumably rare in SRS/MLID and BWS/MLID patients, in contrast to *ZFP57* variants which account for up to 50% in TNDM1 patients [43]. As our investigation was limited to coding sequences, it can be expected that the use of genome-wide NGS-based techniques will provide insight into additional pathogenic variants in noncoding sequences, as well as into the pathoaetiology of MLIDs in general.

In cases with negative WES results, it is currently unclear to what extent either environmental factors or undetected genetic variants contribute to MLID. ART is reported in a significant number of MLID families and is probably a predisposing factor at least for IC2 LOM- or MLID-based BWS, though larger cohorts need to be analysed to confirm this association.

### Study cohort

The study cohort consisted of 36 patients with MLID from 33 families (Fig. 1): 25 patients were referred for BWS testing and eleven patients with features of SRS. They all exhibited MLID identified by MS-MLPA (see below). The BWS/MLID group included two siblings and a pair of concordant monozygotic twins.

Among the MLID patients, 66.7% were female (*n* = 24) and 33.3% male (*n* = 12). The ages of the patients ranged from eleven months to 44.5 years. In 24 families, maternal DNA samples were available.

DNA samples of 27 patients—eight with SRS/MLID and 19 with BWS/MLID—were analysed with a custom targeted methylation sequencing panel (ImprintSeq, [9]). In order to identify genomic maternal effects and foetal variants causing MLID, WES was carried out in 34 patients and 24 mothers. In eight patients and six mothers, data from targeted NGS approaches have been published previously [14–16].

In case clinical data were available, clinical scoring for BWS and SRS was performed based on the scoring systems consented by international consortiums [17, 18].

## Materials and methods

### Methylation-specific multiplex ligation-dependent probe amplification (MS-MLPA)

All patients’ DNA samples were initially tested for CNVs and aberrant methylation of the H19/IGF2:IG-DMR (imprinting centre 1, IC1), and KCNQ1OT1:TSS-DMR (IC2) in 11p15.5 by MS-MLPA (ME030, MRC-Holland, Amsterdam/NL) according to the manufacturer’s manual. In case of patients referred for SRS testing, the assay ME032 addressing the imprinted loci on chromosomes 6, 7, and 14 was used as well.

To identify MLID affecting the further ImpDis-associated iDMRs (PLAGL1:alt-TSS-DMR (PLAGL1), GRB10:alt-TSS-DMR (GRB10), MEST-alt-TSS-DMR (MEST), IC1, IC2, MEG3:TSS-DMR (MEG3), SNURF:TSS-DMR (SNURF), GNASA/B:TSS-DMR (GNASA/B), GNAS-NESP:TSS-DMR (GNAS-NESP), GNAS-AS1:TSS-DMR (GNAS-AS1), GNAS-XL:TSS-DMR (GNAS-XL)) as well as MEG8:Int2-DMR (MEG8) and PEG3:TSS-DMR (PEG3), the assay ME034 was applied. In case of evidence for aberrant methylation at the chromosomal regions 15q11.2, and 20q13.32, the assays ME028 and ME031 were used.

MLID was diagnosed if at least one of these 13 tested iDMRs was affected in addition to the disease-specific iDMR.

### ImprintSeq

For detection of further methylation disturbances including aberrant methylation at non-ImpDis-iDMRs, lymphocyte DNA samples of 19 BWS/MLID patients and eight SRS/MLID were analysed by the targeted methylation sequencing panel ImprintSeq [9]. This assay enables the examination of the CpG methylation status of 63 iDMRs, including the eight ImpDis-associated iDMRs analysed by MS-MLPA. Molecular analyses were conducted according to [9].

The median of the methylation levels across CpGs (median methylation level (MML)) was calculated for each iDMR and each patient. LOM was defined as an MML below the healthy controls’ 3 standard deviations (SD) confidence interval and GOM for those with an MML above 3 SD confidence interval. The MML values of the 70 healthy controls examined by Ochoa et al. were used as a reference [9]. As suggested by Ochoa et al. [9], significant methylation disturbances were classified based on the magnitude of the alteration. Therefore, a cut-off point was calculated by Ochoa et al. which best separates high methylation alterations (HMA; > 0.185) and moderate methylation alterations (MMA; ≤ 0.185)(9).

### Pyrosequencing

As third methylation-specific test, MS pyrosequencing was carried out in case of discrepancies between MS-MLPA and ImprintSeq results for five iDMRs (MEST, IC1, MEG3, SNURF, and PEG3). In brief, 400 ng of DNA was treated with sodium bisulphite by using the EZ DNA Methylation-Gold kit (Zymo, CA, USA). About 40 ng of converted DNA was amplified using the HotStarTaq Master Mix Kit (Qiagen, Hilden, Germany) in a final volume of 25 µl according to the manufacturers protocol. The PCR product was used for quantitative DNA methylation by pyrosequencing on a PyroMark Q96 system with the PyroMark Gold Q96 Reagents Kit (Qiagen, Hilden, Germany). Results were analysed using Pyro Q-CpG software v1.0.9 (Biotage). Information on pyrosequencing primers and assay conditions are available on request.

### Whole-exome sequencing (WES)

WES of 34 patients and 24 mothers was carried out using the IDT xGen Exome Research Panel (v2.0). The libraries were enriched and sequenced on a NextSeq500 Sequencer with 2 × 75 cycles on a high-output flow cell or on a NovaSeq 6000 Sequencer with 2 × 150 cycles on a SP flow cell, respectively. FastQ-files were generated using bcl2fastq2 (Illumina, San Diego, CA, USA). The automated SeqMule pipeline 7 (v1.2.6) was used for alignment and variant calling (GATKLite, 2.3–99). Annotation and bioinformatic prioritisation of variants were carried out using KGGSeq (v1.1, 07/Feb./2019). Variants with a minor allele frequency higher than 0.75% in public databases (i.e. gnomAD (http://gnomad.broadinstitute.org/), EXAC, 1000 GP, ESP) and synonymous variants were excluded. In total, all OMIM (https://www.ncbi.nlm.nih. gov/omim) annotated genes were analysed for variants with putative functional relevance, with a specific focus on genes encoding members of the SCMC (*NLRP2, NLRP5, NLRP7, PADI6, KHDC3L, TLE6*, and *OOEP*) and further candidate genes for MLIDs, e.g. *TRIM28, UHRF1, ZAR1, ARIDA4A, ZFP57,* and Z*NF445*.

## Supplementary Information

**Additional file 1. Table S1** Studies reporting on screening for MLID in ImpDis cohorts. **Table S2** MS-MLPA results in the study population. DNA methylation alterations of 13 imprinted DMRs in 25 BWS/MLID and eleven SRS/MLID patients are shown. Oocyte-derived gDMRs are marked in red, sperm-derived gDMRs in blue. **Table S3** ImprintSeq results. Mean methylation levels (MMLs) per locus and patient. The extent of methylation is shown by a colour gradient (red = high MML; green = low MML). The mean MML of the BWS/MLID and SRS/MLID group and its standard deviation from the control’s MML as well as the frequency of LOMs/GOMs per iDMR are listed below. **Table S4** Application of the molecular MLID criteria proposed by Ochoa et al. (9) in the 27 patients examined by ImprintSeq. **Table S5** Physical positions (CGRCh37/hg19) of the iDMRs analysed by MS-MLPA and ImprintSeq. **Table S6** Discrepancy findings between MS-MLPA and ImprintSeq. In five iDMRs, discrepant results were examined by pyrosequencing. **Table S7** WES results in our cohort. Pathogenic variants detected in NLRP2, NLRP5, and PADI6 in the six families for which data from NGS approaches have been published previously (14-16). The yet unpublished variant in NLRP7 is marked in red. **Table S8** Clinical scores of the BWS/MLID (a) and SRS/MLID (b) patients, leaned on the BWS spectrum (BWSp)-scoring system suggested by Brioude et al. (17) and the Netchine-Harbison clinical scoring system (NH-CSS)(18). The frequency of clinical features of MLID patients is compared with those of single-locus patients of previously published studies (36, 38). **Table S9** Birth parameters of the BWS/MLID and SRS/MLID patients, compared with those of single-locus patients from previously published studies (36, 38). **Table S10** Reproductive history of the MLID mothers. **Table S11** Functional properties of iDMRs with currently unknown clinical relevance but often affected in BWS/MLID or SRS/MLID patients.

## Data Availability

The data reported in the paper are summarised in Additional file table.

## Abbreviations

ART: Assisted reproductive technology
BWS: Beckwith–Wiedemann syndrome
BWSp: BWS spectrum
CNV: Copy number variations
DMML: Differential median methylation level
DMR: Differentially methylated region
gDMR: Germline differentially methylated region
FANCC: FANCC:In61-DMR
GNASA/B: GNASA/B:TSS-DMR
GNAS-AS1: GNAS-AS1:TSS-DMR
GNAS-NESP: GNAS-NESP:TSS-DMR
GNAS-XL: GNAS-XL:TSS-DMR
GOM: Gain of methylation
GRB10: GRB10:alt-TSS-DMR
HMA: High methylation alterations
IC: Imprinting centre
IC1: H19/IGF2:IG-DMR (11p15.5)
IC2: KCNQ1OT1:TSS-DMR (11p15.5)
ICSI: Intracytoplasmic sperm injection
iDMR: Imprinted differentially methylated region
IGF1R: IGF1R:Int2-DMR
ImpDis: Imprinting disorder
IVF: In vitro fertilisation
LOM: Loss of methylation
MEG3: MEG3:TSS-DMR
MEG8: MEG8:Int2-DMR
MEST: MEST-alt-TSS-DMR
MMA: Moderate methylation alterations
MML: Median methylation level
MLID: Multilocus imprinting disturbance
MS-MLPA: Methylation-specific multiplex ligation-dependent probe amplification
NGS: Next-generation sequencing
NH-CSS: Netchine-Harbison Clinical Scoring System
PEG3: PEG3:TSS-DMR
PHP1b: Pseudohypoparathyroidism type 1b
PLAGL1: PLAGL1:alt-TSS-DMR
PWS: Prader–Willi syndrome
SCMC: Subcortical maternal complex
SD: Standard deviation
sDMR: Secondary differentially methylated region
SGA: Small for gestational age
SNU13: SNU13:alt-TSS-DMR
SNURF: SNURF:TSS-DMR
SNV: Small nucleotide variant
SRS: Silver–Russell syndrome
TNDM1: Transient neonatal diabetes mellitus
TS14: Temple syndrome
UPD: Uniparental disomy
VUS: Variant of unknown significance
WES: Whole-exome sequencing
WRB: WRB:alt-TSS-DMR
ZDBF2/GPR1: ZDBF2/GPR1-AS:IG-DMR
ZNF331: ZNF331:alt-TSS-DMR
